# Supplementary material for: Policy addressing suicidality in children and young people: a scoping review protocol
Source: BMJ Open. 2018 Sep 17;8(9):e023153. doi: 10.1136/bmjopen-2018-023153 (PMC6144395; doi:10.1136/bmjopen-2018-023153)
Supplement: Author's manuscript [file bmjopen-2018-023153.draft_revisions.pdf]

BMJ Open

BMJ Open is committed to open peer review. As part of this commitment we make the peer review history of every article we publish publicly available.

When an article is published we post the peer reviewers' comments and the authors' responses online. We also post the versions of the paper that were used during peer review. These are the versions that the peer review comments apply to.

The versions of the paper that follow are the versions that were submitted during the peer review process. They are not the versions of record or the final published versions. They should not be cited or distributed as the published version of this manuscript.

BMJ Open is an open access journal and the full, final, typeset and author-corrected version of record of the manuscript is available on our site with no access controls, subscription charges or pay-per-view fees (http://bmjopen.bmj.com).

If you have any questions on BMJ Open's open peer review process please email info.bmjopen@bmj.com

BMJ Open

Policy addressing suicidality in children and young people: a scoping review protocol.

Journal:	*BMJ Open*
Manuscript ID	bmjopen-2018-023153
Article Type:	Protocol
Date Submitted by the Author:	23-Mar-2018
Complete List of Authors:	Gilmour, Lynne; University of Stirling, NMAPH RU<br>Duncan, Edward; NMAHP-RU<br>Maxwell, Margaret; University of Stirling, Nursing, Midwifery and Allied Health Professions Research Unit
Keywords:	Suicide & self-harm < PSYCHIATRY, Child & adolescent psychiatry < PSYCHIATRY, Health policy < HEALTH SERVICES ADMINISTRATION & MANAGEMENT

SCHOLARONE™
Manuscripts

For peer review only

Policy addressing suicidality in children and young people: a scoping review protocol.

Lynne Gilmour MA (Hons), MRes

NMAPH Research Unit, University of Stirling, Unit 13 Scion House, Innovation Park, Stirling, FK9 4NF.
Contact Email: Lyg1@stir.ac.uk Contact Tel: 01786 466102

Dr Edward Duncan

NMAPH Research Unit, University of Stirling, Unit 13 Scion House, Innovation Park, Stirling, FK9 4NF

Prof Margaret Maxwell

NMAPH Research Unit, University of Stirling, Unit 13 Scion House, Innovation Park, Stirling, FK9 4NF

Word Count: 1861

For peer review only

ABSTRACT

Introduction. Suicide is one of the leading causes of death amongst children and young people globally and a major public health issue. Government policies determine how much recognized health issues are prioritized, and set the context for investment, development and delivery of services. A review of policies concerning children and young people who are suicidal could shed light on the extent that this public health issue is prioritized and highlight examples of best practice in this area. There has never been a review to explore how policy worldwide addresses the specific needs of this vulnerable population. This review will map the key policy documents, and identify their relevance to the review question: How does policy address the treatment and care of Children and Young People who experience suicidality? (International, National (UK), and Local (Scotland)). **Methodology**. Employing scoping review methodological guidance a systematic and transparent approach will be taken. Preliminary searches will facilitate the identification of MeSh terms, Subject headings, and individual database and platform nuances. A full search strategy will be created to search five databases; CINAHL, PsychInfo, Medline, Web of Science, and Cochrane. Government, and other key agency websites (e.g. WHO, UNICEF) will be searched to identify policy documents. The reference lists of identified documents will be checked. A second reviewer will independently screen and cross validate eligible studies for final inclusion. A data extraction template will then be used to extract key information. We will report our findings using narrative synthesis and tabulate findings, by agreed key components. **Ethics and Dissemination**. Ethical approval is not required to conduct a scoping review. We will disseminate the findings through a peer –reviewed publication, and conference presentation.

Registration: This review was has not been registered in any other format.

Key-Words: Suicide, children, adolescents, young people, Policy, Scoping-Review, Review.

STRENGTHS AND LIMITATIONS

- This will be the first scoping review of policy to consider how policy addresses suicidality in CYP, mapping the relevant documents, as well as identifying any gaps.
- Utilising a systematic approach, and methodological guidance (1–3) this review will be rigorous and credible.
- The review will be limited in that it will only include documents written in English.
- Given the iterative nature of searching for policy documents, this review will not capture every available worldwide policy pertaining to suicidal children and young people, but will situate Scottish and UK policy within an international context.

For peer review only

INTRODUCTION

Reducing suicide rates is a global public health priority, and a specific target of the first World Health Organisation (WHO) Mental Health Action Plan (4) which called for a lifespan approach to mental health. Member nations of the WHO were then called to implement national suicide prevention strategies (5). To date this has been achieved in 28 countries with most suicide prevention strategies covering the entire lifespan (6).

Suicide is the second leading cause of death amongst children and young people worldwide (CYP) (7). As suicide prevention strategies have taken a lifespan approach, there is potential for their specific needs to get lost. A recent review highlighted that there were even differences between the presenting issues for children (aged <15 years) compared to adolescents (not defined) who later died by suicide (8). It is widely acknowledged that CYP have different needs to adults, and different health policy and service provision is common place in many countries. Yet little is known about how policy specifically addresses suicidality in children and young people (CYP) and which policies can serve as examples of best practice or innovation in tackling suicide in this population.

To date most systematic reviews in this area have focused upon the effectiveness of interventions, and not the content of policy documents themselves (9–11). Government policies determine how much recognized health issues are prioritized, and set the context for investment, development and delivery of services. However, the gulf that often exists between policies and practice is highlighted by Fortune & Clarkston (12) who state that Child and Adolescent Mental Health services in New Zealand are overwhelmed with referrals and lack the tools and resources to address suicide in young people beyond assessing risk (13).

To develop or evaluate services and interventions for CYP who are suicidal, the policy context within which they are delivered must be taken into account. Despite the development of strategy documents that aim to address suicide, and CYP being identified as a priority population, it is unclear how policy per se addresses the needs of suicidal children and young people specifically. How the

For peer review only

vision expressed in suicide prevention policies translates into the separate government policies that are concerned with addressing the needs of CYP who are suicidal within countries is also unclear.

This review will use scoping review methodology (1–3) to map the relevant available policies worldwide and establish how they address the treatment and care of CYP who are suicidal. The review will enable gaps in policy provision for CYP who are suicidal to be identified and highlight the strategic direction for treating and responding to individual CYP who have either attempted or considered suicide. This review will directly contribute to a larger researcher project that will be conducted in Scotland, concerned with child and adolescent mental health services treatment of CYP who are suicidal.

METHODOLOGY

The scoping review question – **How does policy address the treatment and care of Children and Young People who experience suicidality? (International, National (UK), and Local (Scotland))** was developed using the Joanna Briggs Institute guidance for Umbrella Reviews (3). The search strategy will be systematic in its approach, employing established guidelines (1–3) to inform the methodological process.

Review Aims:

- Identify and map policy documents that relate to children and young people who are suicidal.
- Determine how the policies relate to and address children and young people who are suicidal.
- Establish any gaps within the policies in terms of addressing children who are suicidal.

Search Strategy

The review question was used to generate key words that will be used as search terms (Table 1).

Table 1: Key-word search terms

For peer review only

Concept	Key-words
Children and Young People (5-18yrs)	Child*; "young people"; youth; adolesc*; teen*; paediatric
Suicide	Suicide; suicidal;
Policy	Policy; Procedure; Guidance; Strategy
LIMITS	English Language; Published after 2000.

These search terms will be amended for each of the different databases, and platforms used to include MeSH terms and subject headings. Preliminary searches were conducted to refine the search terms and identify the most appropriate databases and platforms. Four databases will be searched: - CINAHL; Medline; PsychInfo; and The Cochrane Database of Systematic Reviews. The websites of the following key government, statutory and non-statutory agencies will be searched, focusing on post-industrial nations with developed economies in order to identify those with most applicability to the UK: - WHO; UNICEF, UK Government; Scottish Government; ScotPHO; NICE (UK National Institute of Health Care Excellence); National Office of Suicide Prevention (Ireland); Ministry of Health NZ; Australian Government Website; and the Mental Health Commission Canada. Google, and Google Scholar will also be used to identify other policy documents and grey literature. References of identified documents will be checked. Leading experts in the field will be emailed and requested to list key policies as a method of triangulating data collection and ensuring rigor.

Population

Key characteristics of the study population are age, and suicidality. Defining this population is complicated by the variation in what age constitutes being a child or a young person, and by definitions of self-harm to include suicidal intent. The United Nations Convention on the Rights of The Child states anyone under 18 years is a child, The World Health Organisation defines adolescence as age 12-18 years, and young people as 12-24 years, and the United Nations use the term "Youth" to refer to people from aged 12 into their thirties (14) . Given the context of the review

For peer review only

is Scotland, and the Children's Scotland Act (1995) (15) defines a child as anyone under 18yrs, and the NICE guidelines (16) for self-harm relate to children over 8yrs, it was agreed that these parameters would be used. Similarly, the use of definitions of self-harm to include suicide attempts, mean that some papers concerned with self-harm may be relevant to a review of policies relating to suicide. As the phenomenon of interest is suicide, documents that refer solely to non-suicidal self-injury will be excluded, and self-harm will not be used as a search term. The target population of children and young people will include all suicidal CYP who are aged 8-18 years, regardless of gender and ethnicity. Documents that relate solely to adult (>18 years) populations, or solely infant populations (< 5 years), will be excluded. However, policies that have a generic title and age span will be included.

Concept

This review is primarily concerned with establishing what policy documents say in relation to the treatment and care of this population, as well as highlighting any variations or gaps in policy. Prevention activity can be universal (e.g. public health approach aimed at everyone), selected (targeted at high-risk groups) and indicated (at the individual treatment or intervention level.). This review is concerned with identifying policy that includes indicated prevention activity: specifically, about the treatment and care for children and young people who are suicidal.

Context

The reviewing authors are based in Scotland (UK) and this review will contribute to a larger study being conducted there. The relevance and applicability of each review to Scotland and the UK, will be assessed and coded using the following criteria:

A. Directly relevant to Scotland and the UK
B. Includes non-UK studies, but the context / population group would apply equally to UK settings
C. Includes non-UK studies that are clearly not relevant to UK settings

For peer review only

Types of Sources

All relevant national, and international policy documents will be included. Local policy documents will refer to Scotland. Individual regions and states may have they their own guidelines, as will individual organisations as part of their workforce policies and procedures, however these should reflect the national priorities and guidance. Although there are subtle differences in defining the terms 'strategy' and 'policy' they are often used interchangeably, and some countries now also use the term "framework" to outline a national approach. For the purposes of this review, policy documents can include Policies, Policy Guidance, Strategies, Codes of Conduct, National Service Frameworks, Practice Guidance, White and Green Papers (17) . Reviews of policy documents centred on CYP who are suicidal will also be included to support what is known in this area.

Screening

Identified policies and documents will be listed in an excel workbook and screened against agreed inclusion and exclusion criteria (Table 2.), first by title and executive summary or abstract (by two authors independently), and then in full text (by author one, with 50% cross validated by a second author). Any disagreements will be discussed with a third author who will act as mediator. All discussion and agreements will be recorded. A PRISMA diagram (18) will be used to record and illustrate the search process.

Table 2: Search Criteria

Inclusion	Exclusion
About children less than 18yrs and over 8yrs.	Not in English
About suicide OR Uses a definition of self-harm to include suicidal behavior.	Published before 2000
National Policy Documents / Strategies	Solely about Non Suicidal Self Injury
About National Policy / Strategy Documents	Solely about universal and selective prevention

For peer review only - http://bmjopen.bmj.com/site/about/guidelines.xhtml

Reviews of Policy	Solely about a population of adults 18>
Most recent version of Policy Document or Strategy	Solely about a population of children <8yrs
Directly relevant to Scotland only OR<br>Relevant to the UK OR Includes non-UK studies, but the context / population group would apply equally to UK settings	Primary Studies
	Includes non-UK studies that are clearly not relevant to UK settings
	Organisational policies e.g. at service level
	Previous version of policy where newer version is available for inclusion.

Data Extraction

A data extraction template will be developed that reflects the research question and the aims of the review. It will include gathering information regarding the key-characteristics of the policy document, such as year, country, type of policy etc. as well as specifics about the policy approach and detail of how it pertains to suicidal CYP. One reviewer (LG) will complete data extraction, with a second reviewer cross validating through a process of independently extracting data (25%). Any disagreements or inconsistencies in the extracted data will be resolved via discussion and will involve the third reviewer.

ETHICS AND DISSEMINATION

No Ethical approval is required to conduct a review.

Charting the data & presenting the findings

Review findings will be presented in a summary table with headings that will reflect the research question and key concepts that emerge, organised by categories such as country, and type of document. Narrative will be used to provide a descriptive overview of the included policy and

For peer review only

research, and indicate any identified gaps. Lay and executive summaries will be produced to make the findings relevant and accessible to the public, practitioners and policy makers. The findings will be widely disseminated through a peer reviewed publication and conference presentations.

Suicide amongst children and young people is a global public health concern. This review will be the first to identify and map international policy pertaining to the treatment and care of CYP who are suicidal.

Acknowledging the iterative process involved in identifying policy documents, and potential language barriers, this review will not capture every policy pertaining to suicidal children and young people across the globe. However, by applying a systematic and well-defined approach this review will robustly and reliably summarise and map the key policy documents. It will then describe how these policies address, or perhaps do not address the treatment and care needs of children and young people who are suicidal. It will provide invaluable knowledge for future policy makers, researchers and practitioners.

ACKNOWELDGEMENTS & COMPETING INTERESTS

LG is an ESRC funded PhD student. No other funding was sought for this Scoping Review. There are no competing interests. Contribution was made by Maya Jeffery, Faculty of Health Sciences Librarian at the University of Stirling, who assisted in the identification of key search terms, and appropriate databases. LG led on development of the Protocol and all authors contributed to writing of the paper.

For peer review only

REFERENCES

1. Arksey H, O 'Malley L. Scoping Studies: Towards a Methodological Framework. Int J Soc Res Methodol [Internet]. 2005;8(1):19–32. Available from: http://journalsonline.tandf.co.uk/%0Ahttp://www.journalsonline.tandf.co.uk/openurl.asp?genre=article&eissn=1464-5300&volume=8&issue=1&spage=19

2. Levac D, Colquhoun H, O'Brien KK. Scoping studies: advancing the methodology. Implement Sci [Internet]. 2010;5(1):69. Available from: http://implementationscience.biomedcentral.com/articles/10.1186/1748-5908-5-69

3. The Joanna Briggs Institute. Reviewers' Manual. 2015; Available from: www.joannabriggs.org

4. WHO. Mental Health Action Plan 2013 - 2020 [Internet]. 2013 [cited 2018 Mar 12]. Available from: http://apps.who.int/iris/bitstream/10665/89966/1/9789241506021_eng.pdf?ua=1

5. Robinson J, Hetrick SE, Martin C. Preventing suicide in young people: Systematic review. Aust N Z J Psychiatry [Internet]. 2011;45(1):3–26. Available from: http://publicaciones.umh.es/docview/837458952?accountid=28939%5Cnhttp://onlinelibrary.wiley.com/o/cochrane/cldare/articles/DARE-12011000804/frame.html

6. WHO. WHO | Suicide prevention [Internet]. WHO. World Health Organization; 2018 [cited 2017 Dec 18]. Available from: http://www.who.int/mental_health/suicide-prevention/en/

7. WHO. Preventing suicide. CMAJ. 2014;143(7):609–10.

8. Soole R, Kõlves K, De Leo D. Suicide in Children: A Systematic Review. Arch Suicide Res [Internet]. 2015 Jul 3 [cited 2018 Jan 10];19(3):285–304. Available from: http://www.tandfonline.com/doi/full/10.1080/13811118.2014.996694

9. Bennett K, Rhodes AE, Duda S, Cheung AH, Manassis K, Links P, et al. A Youth Suicide Prevention Plan for Canada: A Systematic Review of Reviews. Can J Psychiatry CanJPsychiatry [Internet]. 2015 [cited 2018 Mar 12];6060(66):245–57. Available from: www.TheCJP.ca

10. Scott A, Guo B. For which strategies of suicide prevention is there evidence of effectiveness? DECISION SUPPORT TECHNIQUE EVIDENCE BASED PRACTICE HARM REDUCTION PREVENTIVE HEALTH SERVICES RISK REDUCTION BEHAVIOR SUICIDE – PREVENTION AND CONTROL SUICIDE, ATTEMPTED – PREVENTION AND CONTROL. 2012 [cited 2018 Mar 12]; Available from: http://www.euro.who.int/__data/assets/pdf_file/0003/168843/HEN-Suicide-Prevention-synthesis-report.pdf

11. Zalsman G, Hawton K, Wasserman D, van Heeringen K, Arensman E, Sarchiapone M, et al. Suicide prevention strategies revisited: 10-year systematic review. The Lancet Psychiatry [Internet]. 2016 Jul [cited 2018 Mar 12];3(7):646–59. Available from: http://www.ncbi.nlm.nih.gov/pubmed/27289303

12. Fortune S, Clarkson H. The Role of Child and Adolescent Mental Health Services in Suicide Prevention in New Zealand. Australas Psychiatry [Internet]. 2006 Dec 25 [cited 2018 Mar 12];14(4):369–73. Available from: http://www.ncbi.nlm.nih.gov/pubmed/17116074

13. Fortune S, Clarkson H. The role of child and adolescent mental health services in suicide prevention in New Zealand. 2006 [cited 2018 Feb 2]; Available from: http://journals.sagepub.com.ezproxy.stir.ac.uk/doi/pdf/10.1080/j.1440-1665.2006.02306.x

For peer review only

14. WHO. Adolescence: a period needing special attention - recognizing-adolescence [Internet]. 2018 [cited 2018 Feb 5]. Available from: http://apps.who.int/adolescent/second-decade/section2/page1/recognizing-adolescence.html

15. Scottish Government. Children (Scotland) Act 1995 [Internet]. Statute Law Database; 1995. Available from: https://www.legislation.gov.uk/ukpga/1995/36/contents

16. NICE. Self-harm in o Self-harm in ov ver 8s: short-term er 8s: short-term management and pre management and prev vention of ention of recurrence recurrence Clinical guideline. 2004;(July).

17. Stanley N, Cox P, Ashcroft J, Bailey S, Carter B, Carter Y, et al. Parental mental health and child welfare: reviews of policy and professional education [Internet]. 2009 [cited 2018 Mar 12]. Available from: https://www.scie.org.uk/publications/guides/guide30/files/FullResearchReview.pdf?res=true

18. Moher D, Shamseer L, Clarke M, Ghersi D, Liberati A, Petticrew M, et al. Preferred reporting items for systematic review and meta-analysis protocols (PRISMA-P) 2015 statement. Syst Rev. 2015;4(1).

For peer review only

BMJ Open

Policy addressing suicidality in children and young people: a scoping review protocol.

Journal:	*BMJ Open*
Manuscript ID	bmjopen-2018-023153.R1
Article Type:	Protocol
Date Submitted by the Author:	27-Jun-2018
Complete List of Authors:	Gilmour, Lynne; University of Stirling, NMAPH RU<br>Duncan, Edward; NMAHP-RU<br>Maxwell, Margaret; University of Stirling, Nursing, Midwifery and Allied Health Professions Research Unit
**Primary Subject Heading**:	Health policy
Secondary Subject Heading:	Mental health, Global health, Health policy, Paediatrics
Keywords:	Suicide & self-harm < PSYCHIATRY, Child & adolescent psychiatry < PSYCHIATRY, Health policy < HEALTH SERVICES ADMINISTRATION & MANAGEMENT

SCHOLARONE™
Manuscripts

Policy addressing suicidality in children and young people: a scoping review protocol.

Lynne Gilmour MA (Hons), MRes

NMAPH Research Unit, University of Stirling, Unit 13 Scion House, Innovation Park, Stirling, FK9 4NF. Contact Email: Lyg1@stir.ac.uk Contact Tel: 01786 466102

Dr Edward Duncan

NMAPH Research Unit, University of Stirling, Unit 13 Scion House, Innovation Park, Stirling, FK9 4NF

Prof Margaret Maxwell

NMAPH Research Unit, University of Stirling, Unit 13 Scion House, Innovation Park, Stirling, FK9 4NF

Word Count: 1919

For peer review only

For peer review only - http://bmjopen.bmj.com/site/about/guidelines.xhtml

ABSTRACT

Introduction. Suicide is one of the leading causes of death amongst children and young people globally and a major public health issue. Government policies determine how much recognized health issues are prioritized, and set the context for investment, development and delivery of services. A review of policies concerning children and young people who are suicidal could shed light on the extent that this public health issue is prioritized and highlight examples of best practice in this area. There has never been a review to explore how policy worldwide addresses the specific needs of this vulnerable population. This review will map the key policy documents, and identify their relevance to the review question: How does policy address the treatment and care of Children and Young People who experience suicidality? (International, National (UK), and Local (Scotland)). **Methodology**. Employing scoping review methodological guidance a systematic and transparent approach will be taken. Preliminary searches will facilitate the identification of MeSh terms, Subject headings, and individual database and platform nuances. A full search strategy will be created to search five databases; CINAHL, PsychInfo, Medline, Web of Science, and Cochrane. Government, and other key agency websites (e.g. WHO, UNICEF) will be searched to identify policy documents. The reference lists of identified documents will be checked. A second reviewer will independently screen and cross validate eligible studies for final inclusion. A data extraction template will then be used to extract key information. We will report our findings using narrative synthesis and tabulate findings, by agreed key components. **Ethics and Dissemination**. Ethical approval is not required to conduct a scoping review. We will disseminate the findings through a peer –reviewed publication, and conference presentation.

Registration: This review was has not been registered in any other format.

Key-Words: Suicide, children, adolescents, young people, Policy, Scoping-Review, Review.

STRENGTHS AND LIMITATIONS

- This will be the first scoping review of policy to consider how policy addresses suicidality in CYP, mapping the relevant documents, as well as identifying any gaps.
- Utilising a systematic approach, and methodological guidance this review will be rigorous and credible.
- The review will be limited in that it will only include documents written in English.
- Given the iterative nature of searching for policy documents, this review will not capture every available worldwide policy pertaining to suicidal children and young people, but will situate Scottish and UK policy within an international context.

For peer review only - http://bmjopen.bmj.com/site/about/guidelines.xhtml

For peer review only

INTRODUCTION

Reducing suicide rates is a global public health priority, and a specific target of the first World Health Organisation (WHO) Mental Health Action Plan (1) which called for a lifespan approach to mental health. Member nations of the WHO were then called to implement national suicide prevention strategies (2). To date this has been achieved in 28 countries with most suicide prevention strategies covering the entire lifespan (3).

Suicide is the second leading cause of death amongst children and young people worldwide (4), and in the UK is the main cause of death (5). A recent UK report notes that 14% of deaths amongst 10-19yr old's in England and Wales were caused by suicide, and that having an undetermined cause of death recorded was much higher amongst under 16yr old's meaning that potentially this number could be much higher (5).

As suicide prevention strategies have taken a lifespan approach, there is potential for the specific needs of children and young people to become lost. A recent review highlighted that there were even differences between the presenting issues for children (aged <15 years) compared to adolescents (not defined) who later died by suicide (6). It is widely acknowledged that children and young people have different needs to adults, and different health policy and service provision is common place in many countries. Yet little is known about how policy specifically addresses suicidality in children and young people and which policies can serve as examples of best practice or innovation in tackling suicide in this population.

To date most systematic reviews in this area have focused upon the effectiveness of interventions, and not the content of policy documents themselves (7-9). Government policies determine how much recognized health issues are prioritized, and set the context for investment, development and delivery of services. However, the gulf that often exists between policies and practice is highlighted by Fortune & Clarkston (10) who state that Child and Adolescent Mental Health services in New

For peer review only - http://bmjopen.bmj.com/site/about/guidelines.xhtml

Zealand are overwhelmed with referrals and lack the tools and resources to address suicide in young people beyond assessing risk (10).

To develop or evaluate services and interventions for children and young people who are suicidal, the policy context within which they are delivered must be taken into account. Despite the development of strategy documents that aim to address suicide, and children and young people being identified as a priority population, it is unclear how policy per se addresses the needs of suicidal children and young people specifically. How the vision expressed in suicide prevention policies translates into the separate government policies that are concerned with addressing the needs of children and young people who are suicidal within countries is also unclear.

This review will use scoping review methodology (11-13) to map the relevant available policies worldwide and establish how they address the treatment and care of children and young people who are suicidal. The review will enable gaps in policy provision for children and young people who are suicidal to be identified and highlight the strategic direction for treating and responding to individual children and young people who have either attempted or considered suicide. This review will directly contribute to a larger researcher project that will be conducted in Scotland, concerned with child and adolescent mental health services treatment of children and young people who are suicidal.

METHODOLOGY

The scoping review question – **How does policy address the treatment and care of Children and Young People who experience suicidality? (International, National (UK), and Local (Scotland))** was developed using the Joanna Briggs Institute guidance for Umbrella Reviews (13). The search strategy will be systematic in its approach, employing established guidelines (11–13) to inform the methodological process.

Review Aims:

For peer review only - http://bmjopen.bmj.com/site/about/guidelines.xhtml

- Identify and map policy documents that relate to children and young people who are suicidal.
- Determine how the policies relate to and address children and young people who are suicidal.
- Establish any gaps within the policies in terms of addressing children who are suicidal.
- Explore the potential need for any future thematic or discursive analysis of how policy deals with children and young people who are suicidal.

Search Strategy

The review question was used to generate key words that will be used as search terms (Table 1).

Table 1: Key-word search terms

Concept	Key-words
Children and Young People (5-18yrs)	Child*; “young people”; youth; adolesc*; teen*; paediatric
Suicide	Suicide; suicidal;
Policy	Policy; Procedure; Guidance; Strategy
LIMITS	English Language; Published after 2000.

These search terms will be amended for each of the different databases, and platforms used to include MeSH terms and subject headings. Preliminary searches were conducted to refine the search terms and identify the most appropriate databases and platforms. Four databases will be searched: - CINAHL; Medline; PsychInfo; and The Cochrane Database of Systematic Reviews. The websites of the following key government, statutory and non-statutory agencies will be searched, focusing on post-industrial nations with developed economies in order to identify those with most applicability to the UK: - WHO; UNICEF, UK Government; Scottish Government; ScotPHO; NICE (UK National Institute of Health Care Excellence); National Office of Suicide Prevention (Ireland); Ministry of Health NZ; Australian Government Website; and the Mental Health Commission Canada. Google, and Google

For peer review only - http://bmjopen.bmj.com/site/about/guidelines.xhtml

Scholar will also be used to identify other policy documents and grey literature. References of identified documents will be checked. Leading experts in the field will be emailed and requested to list key policies as a method of triangulating data collection and ensuring rigor.

Population

Key characteristics of the study population are age, and suicidality. Defining this population is complicated by the variation in what age constitutes being a child or a young person, and by definitions of self-harm to include suicidal intent. The United Nations Convention on the Rights of The Child states anyone under 18 years is a child, The World Health Organisation defines adolescence as age 12-18 years, and young people as 12-24 years, and the United Nations use the term "Youth" to refer to people from aged 12 into their thirties (14) . Given the context of the review is Scotland, and the Children's Scotland Act (1995) (15) defines a child as anyone under 18yrs, and the NICE guidelines (16) for self-harm relate to children over 8yrs, it was agreed that these parameters would be used. Similarly, the use of definitions of self-harm to include suicide attempts, mean that some papers concerned with self-harm may be relevant to a review of policies relating to suicide. As the phenomenon of interest is suicide, documents that refer solely to non-suicidal self-injury will be excluded, and self-harm will not be used as a search term. The target population of children and young people will include all suicidal children and young people who are aged 8-18 years, regardless of gender and ethnicity. Documents that relate solely to adult (>18 years) populations, or solely infant populations (< 5 years), will be excluded. However, policies that have a generic title and age span will be included.

Concept

This review is primarily concerned with establishing what policy documents say in relation to the treatment and care of this population, as well as highlighting any variations or gaps in policy. Prevention activity can be universal (e.g. public health approach aimed at everyone), selected

For peer review only - http://bmjopen.bmj.com/site/about/guidelines.xhtml

(targeted at high-risk groups) and indicated (at the individual treatment or intervention level.). This review is concerned with identifying policy that includes indicated prevention activity: specifically, about the treatment and care for children and young people who are suicidal.

Context

The reviewing authors are based in Scotland (UK) and this review will contribute to a larger study being conducted there. Although Scotland remains part of the UK, health has always been a devolved issue and the NHS policy across the two nations is markedly different. The relevance and applicability of each policy document to Scotland and the UK, will be assessed and coded using the following criteria:

A. Directly relevant to Scotland and the UK
B. Includes non-UK studies, but the context / population group would apply equally to UK settings
C. Includes non-UK studies that are clearly not relevant to UK settings

Types of Sources

All relevant national, and international policy documents will be included. Local policy documents will refer to Scotland. Individual regions and states may have they their own guidelines, as will individual organisations as part of their workforce policies and procedures, however these should reflect the national priorities and guidance. Although there are subtle differences in defining the terms 'strategy' and 'policy' they are often used interchangeably, and some countries now also use the term "framework" to outline a national approach. For the purposes of this review, policy documents can include Policies, Policy Guidance, Strategies, Codes of Conduct, National Service Frameworks, Practice Guidance, White and Green Papers (17). Reviews of policy documents centred on CYP who are suicidal will also be included to support what is known in this area.

For peer review only

For peer review only - http://bmjopen.bmj.com/site/about/guidelines.xhtml

Screening

Identified policies and documents will be listed in an excel workbook and screened against agreed inclusion and exclusion criteria (Table 2.), first by title and executive summary or abstract (by two authors independently), and then in full text (by author one, with a random sample of 50% cross validated by a second author). Any disagreements will be discussed with a third author who will act as mediator. All discussion and agreements will be recorded. A PRISMA diagram (18) will be used to record and illustrate the search process.

Table 2: Search Criteria

Inclusion	Exclusion
About children less than 18yrs and over 8yrs.	Not in English
About suicide OR Uses a definition of self-harm to include suicidal behavior.	Published before 2000
National Policy Documents / Strategies	Solely about Non Suicidal Self Injury
About National Policy / Strategy Documents	Solely about universal and selective prevention
Reviews of Policy	Solely about a population of adults 18>
Most recent version of Policy Document or Strategy	Solely about a population of children <8yrs
Directly relevant to Scotland only OR<br>Relevant to the UK OR Includes non-UK studies, but the context / population group would apply equally to UK settings	Primary Studies
	Includes non-UK studies that are clearly not relevant to UK settings
	Organisational policies e.g. at service level
	Previous version of policy where newer version is available for inclusion.

For peer review only - http://bmjopen.bmj.com/site/about/guidelines.xhtml

Data Extraction

A data extraction template will be developed that reflects the research question and the aims of the review. It will include gathering information regarding the key-characteristics of the policy document, such as year, country, type of policy etc. as well as specifics about the policy approach and detail of how it pertains to suicidal children and young people. One reviewer (LG) will complete data extraction, with a second reviewer cross validating through a process of independently extracting data (25%) from a random sample of included policies. Any disagreements or inconsistencies in the extracted data will be resolved via discussion and will involve the third reviewer.

PATIENT AND PUBLIC INVOLVEMENT

The public and patients were not consulted during the development of this scoping review protocol.

ETHICS AND DISSEMINATION

No Ethical approval is required to conduct a review.

Charting the data & presenting the findings

Review findings will be presented in a summary table with headings that will reflect the research question and key concepts that emerge, organised by categories such as country, and type of document. Scoping reviews are used to provide a broad overview rather than in-depth analysis of a topic area and can often help to establish the feasibility of a future systematic review or qualitative evidence synthesis. Narrative will be used to provide a descriptive overview of the included policy and research and indicate any identified gaps. Lay and executive summaries will be produced to make the findings relevant and accessible to the public, practitioners, and policy makers. The findings will be widely disseminated through a peer reviewed publication and conference presentations.

For peer review only

For peer review only - http://bmjopen.bmj.com/site/about/guidelines.xhtml

Suicide amongst children and young people is a global public health concern. This review will be the first to identify and map international policy pertaining to the treatment and care of CYP who are suicidal.

Acknowledging the iterative process involved in identifying policy documents, and potential language barriers, this review will not capture every policy pertaining to suicidal children and young people across the globe. However, by applying a systematic and well-defined approach this review will robustly and reliably summarise and map the key policy documents. It will then describe how these policies address, or perhaps do not address the treatment and care needs of children and young people who are suicidal. It will provide invaluable knowledge for future policy makers, researchers and practitioners.

For peer review only

For peer review only - http://bmjopen.bmj.com/site/about/guidelines.xhtml

REFERENCES

1. WHO. Mental Health Action Plan 2013 - 2020 [Internet]. 2013 [cited 2018 Mar 12]. Available from: http://apps.who.int/iris/bitstream/10665/89966/1/9789241506021_eng.pdf?ua=1

2. Robinson J, Hetrick SE, Martin C. Preventing suicide in young people: Systematic review. Aust N Z J Psychiatry [Internet]. 2011;45(1):3–26. Available from: http://publicaciones.umh.es/docview/837458952?accountid=28939%5Cnhttp://onlinelibrary.wiley.com/o/cochrane/cldare/articles/DARE-12011000804/frame.html

3. WHO. WHO | Suicide prevention [Internet]. WHO. World Health Organization; 2018 [cited 2017 Dec 18]. Available from: http://www.who.int/mental_health/suicide-prevention/en/

4. WHO. Preventing suicide. CMAJ. 2014;143(7):609–10.

5. NCISH. Suicide by children and young people. National Confidential Inquiry into Suicide and Homicide by People with Mental Illness. 2017, Manchester: University of Manchester.

6. Soole R, Kõlves K, De Leo D. Suicide in Children: A Systematic Review. Arch Suicide Res [Internet]. 2015 Jul 3 [cited 2018 Jan 10];19(3):285–304. Available from: http://www.tandfonline.com/doi/full/10.1080/13811118.2014.996694

7. Bennett K, Rhodes AE, Duda S, Cheung AH, Manassis K, Links P, et al. A Youth Suicide Prevention Plan for Canada: A Systematic Review of Reviews. Can J Psychiatry CanJPsychiatry [Internet]. 2015 [cited 2018 Mar 12];6060(66):245–57. Available from: www.TheCJP.ca

8. Scott A, Guo B. For which strategies of suicide prevention is there evidence of effectiveness? DECISION SUPPORT TECHNIQUE EVIDENCE BASED PRACTICE HARM REDUCTION PREVENTIVE HEALTH SERVICES RISK REDUCTION BEHAVIOR SUICIDE – PREVENTION AND CONTROL SUICIDE, ATTEMPTED – PREVENTION AND CONTROL. 2012 [cited 2018 Mar 12]; Available from: http://www.euro.who.int/__data/assets/pdf_file/0003/168843/HEN-Suicide-Prevention-synthesis-report.pdf

9. Zalsman G, Hawton K, Wasserman D, van Heeringen K, Arensman E, Sarchiapone M, et al. Suicide prevention strategies revisited: 10-year systematic review. The Lancet Psychiatry [Internet]. 2016 Jul [cited 2018 Mar 12];3(7):646–59. Available from: http://www.ncbi.nlm.nih.gov/pubmed/27289303

10. Fortune S, Clarkson H. The Role of Child and Adolescent Mental Health Services in Suicide Prevention in New Zealand. Australas Psychiatry [Internet]. 2006 Dec 25 [cited 2018 Mar 12];14(4):369–73. Available from: http://www.ncbi.nlm.nih.gov/pubmed/17116074

11. Arksey H, O ’Malley L. Scoping Studies: Towards a Methodological Framework. Int J Soc Res Methodol [Internet]. 2005;8(1):19–32. Available from: http://journalsonline.tandf.co.uk/%0Ahttp://www.journalsonline.tandf.co.uk/openurl.asp?genre=article&eissn=1464-5300&volume=8&issue=1&spage=19

12. Levac D, Colquhoun H, O'Brien KK. Scoping studies: advancing the methodology. Implement Sci [Internet]. 2010;5(1):69. Available from: http://implementationscience.biomedcentral.com/articles/10.1186/1748-5908-5-69

13. The Joanna Briggs Institute. Reviewers' Manual. 2015; Available from: www.joannabriggs.org

For peer review only - http://bmjopen.bmj.com/site/about/guidelines.xhtml

14. WHO. Adolescence: a period needing special attention - recognizing-adolescence [Internet]. 2018 [cited 2018 Feb 5]. Available from: http://apps.who.int/adolescent/second-decade/section2/page1/recognizing-adolescence.html

15. Scottish Government. Children (Scotland) Act 1995 [Internet]. Statute Law Database; 1995. Available from: https://www.legislation.gov.uk/ukpga/1995/36/contents

16. NICE. Self-harm in o Self-harm in over 8s: short-term 8s: short-term management and pre-management and prevention of recurrence Clinical guideline. 2004;(July).

17. Stanley N, Cox P, Ashcroft J, Bailey S, Carter B, Carter Y, et al. Parental mental health and child welfare: reviews of policy and professional education [Internet]. 2009 [cited 2018 Mar 12]. Available from: https://www.scie.org.uk/publications/guides/guide30/files/FullResearchReview.pdf?res=true

18. Moher D, Shamseer L, Clarke M, Ghersi D, Liberati A, Petticrew M, et al. Preferred reporting items for systematic review and meta-analysis protocols (PRISMA-P) 2015 statement. Syst Rev. 2015; 4 (1).

ACKNOWLEDGEMENTS & COMPETING INTERESTS

There are no competing interests. Contribution was made by Maya Jeffery, Faculty of Health Sciences Librarian at the University of Stirling, who assisted in the identification of key search terms, and appropriate databases. LG led on development of the Protocol and all authors contributed to writing of the paper.

FUNDING

LG is an ESRC funded PhD student. No other funding was received for this Scoping Review from any funding agency in the public, commercial or not-for-profit sectors.

AUTHORS CONTRIBUTIONS

The idea for the protocol was conceived by LG. The search strategy and parameters of the review were established and agreed by LG, ED, and MM equally. The protocol was first drafted by LG. It was then edited by re-drafted by ED and MM. The manuscript was then edited and redrafted by LG, ED and MM following reviewers' comments. All authors have contributed to and approved the final version of the manuscript.

For peer review only

For peer review only - http://bmjopen.bmj.com/site/about/guidelines.xhtml
